# Selective Growth and Contact Gap-Fill of Low Resistivity Si via Microwave Plasma-Enhanced CVD

**DOI:** 10.3390/mi10100689

**Published:** 2019-10-12

**Authors:** Youngwan Kim, Myoungwoo Lee, Youn-Jea Kim

**Affiliations:** 1 Graduate School of Mechanical Engineering, Sungkyunkwan University, Suwon 16419, Korea; giogist@skku.edu (Y.K.); lmw1238@skku.edu (M.L.); 2 School of Mechanical Engineering, Sungkyunkwan University, Suwon 16419, Korea

**Keywords:** microwave plasma, gap-fill process, selective Si growth

## Abstract

Low resistivity polycrystalline Si could be selectively grown in the deep (~200 nm) and narrow patterns (~20 nm) of 20 nm pitch design rule DRAM (Dynamic Random Access Memory) by microwave plasma-enhanced chemical vapor deposition (MW-CVD). We were able to achieve the high phosphorus (CVD gap-fill in a large electrical contact area which does is affected by line pitch size) doping concentration (>2.5 × 10^21^ cm^−3^) and, thus, a low resistivity by adjusting source gas (SiH_4_, H_2_, PH_3_) decomposition through MW-CVD with a showerhead controlling the decomposition of source gases by using two different gas injection paths. In this study, a selective growth mechanism was applied by using the deposition/etch cyclic process to achieve the bottom–up process in the L-shaped contact, using H_2_ plasma that simultaneously promoted the deposition and the etch processes. Additionally, the cyclic selective growth technique was set up by controlling the SiH_4_ flow rate. The bottom-up process resulted in a uniform doping distribution, as well as an excellent filling capacity without seam and center void formation. Thus, low contact resistivity and higher transistor on-current could be achieved at a high and uniform phosphorus (P)-concentration. Compared to the conventional thermal, this method is expected to be a strong candidate for the complicated deep and narrow contact process.

## 1. Introduction

Since the design rule of the DRAM (Dynamic Random Access Memory) device and its contact size has continuously shrunk and its aspect ratio of contact has steadily increased, the traditional doped Si gap-fill method by thermal chemical vapor deposition (CVD) may no longer be extendable to the deep and narrow contact hole with a complicated design, e.g., the L-shaped gate buried contact (gBC). The main reasons for this limitation are seam and void formation near the middle of the contact area, which results from higher doping concentrations. Numerous alternative solutions for these difficulties have been proposed, such as thermal CVD with an amino-silane seed layer [1], Si implantation after Si etch-back, flowable CVD and laser-induced epitaxial growth [2]. However, all of these methods cannot meet simultaneously requirements. Considering these needs and difficulties, bottom–up growth using a selective growth technique is a candidates for Si gap-filling without void and seam formation. However, the dopant incorporation is limited due to growth rate reduction and crystal quality degradation, especially n-type dopants [3,4]. Recently, the microwaves radiating from a radial-line slot antenna (RLSA) were used at a CVD system for oxidation and nitridation processes [5,6,7]. The RLSA plasma source has advantages such as a low electron temperature, high electron density, high-power handling capability, and aperture field uniformity [5,6].

In this study, we applied a microwave plasma-enhanced CVD (MW-CVD) system with an RLSA for the growth of high doped n-type polycrystalline Si. The bottom-up gap-filling by selective growth with the high phosphorus (P) concentration was achieved by a showerhead that allowed us to control the source gas decomposition.

## 2. Experimental Details

**Figure 1:**
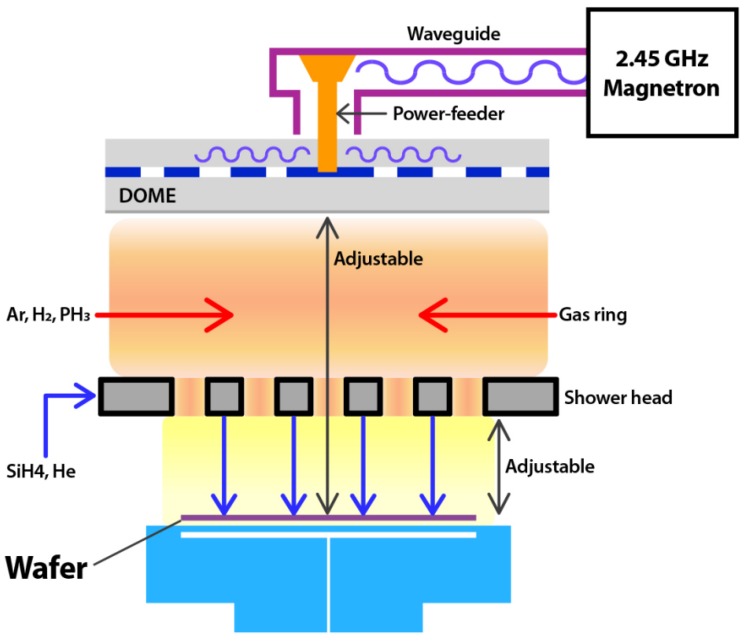
Schematic of the microwave plasma reactor.

All the experiments were conducted in a cold wall type MW-CVD, as shown in Figure 1. The 2.45 GHz microwave which radiated from an RLSA was introduced into the process chamber through a dielectric plate. High efficiency power transformation from the microwave to the plasma with low reflection power was achieved in the microwave power range of 2400–3800 W. We could control the decomposition of source gases by using two different gas injection paths. One was the gas ring that fed gases at the upper region of the chamber, and the other was the showerhead that fed gases at the lower region of the chamber, wherein a low electron temperature was maintained [8]. To generate the plasma, Ar and H_2_ gases were injected with the gas ring with flow rates of 100–250 and 100–1000 SCCM, respectively. PH_3_ (15% diluted in H_2_) was also injected with the gas ring to achieve a high decomposition rate and, thus, a high doping concentration with the flow rate in the range of 0–20 SCCM. SiH_4_ was injected directly at the wafer surface from the showerhead gas holes with the flow rate of 0–20 SCCM. Plasma was generated at a pressure of under 500 mTorr, and we kept the pressure of 30–40 mTorr at the growth step by evacuating a turbo molecular pump (3 m^3^/hr) with a base pressure of 1 × 10^−7^ Torr. The growth temperature varied between 350 and 520 °C. The growth selectivity was confirmed by using 100 nm-thick thermal grown oxide wafers and bare Si wafers. Prior to Si gap-filling at the 20 nm pitch gBC pattern, the wafers were treated by the plasma NF_3_ clean and loaded into the vacuum load-lock within 60 min in order to remove the native oxide and maintain the H-passivated Si surface. Grown Si structures were analyzed by X-ray diffraction (XRD) (Bruker, Billerica, MA, USA) with Cu Kα1 radiation. In-line X-ray fluorescence (XRF) (Bruker, Billerica, MA, USA) was used for measuring the P concentration in the layer, which was confirmed by secondary ion mass spectrometry (SIMS). A Secondary electron microscope (SEM) and cross-section transmission electron microscope (X-TEM) analyses were used to observe the profile, such as seam, void existence, and contact epitaxial growth (CEG) formation between the polycrystalline Si and the active Si interface. To identify the doping characteristics in the pattern, we performed two-dimensional energy dispersive spectroscopy (EDS) mapping.

## 3. Results and Discussion

**Figure 2:**
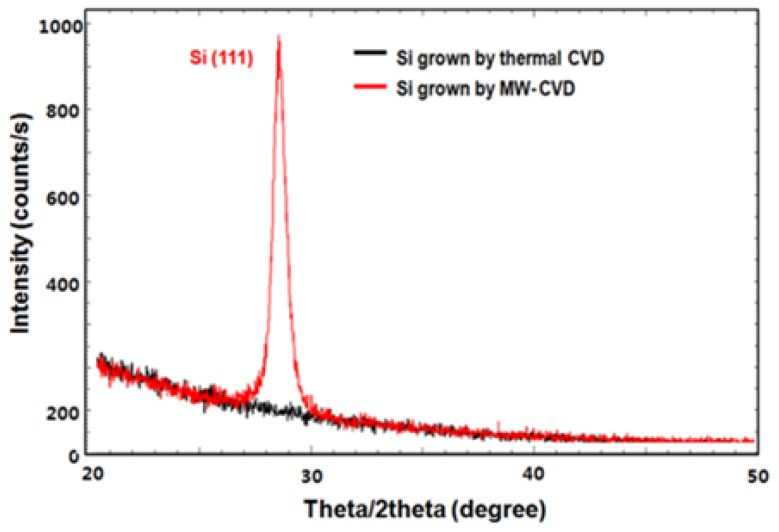
X-ray diffraction (XRD) rocking curves of the Si layers grown by thermal chemical vapor deposition (CVD) (580 °C; black) and microwave plasma-enhanced chemical vapor deposition (MW-CVD) (430 °C; red).

Figure 2 shows XRD results of the Si grown on SiO_2_ by thermal CVD and MW-CVD. The black line shows that no peak appeared in the range of the X-ray scan, representing amorphous Si due to the low growth temperature of 580 °C. Conversely, a high intensity Si (1 1 1) peak appeared in the X-ray scan of the Si grown by MW-CVD (red line), representing crystallized Si, although the growth temperature was low (430 °C) compared to that of the thermal CVD. In the CVD, as the energy of electrons in the plasma varied from 0 to several 10 s volts (eV), the ground-state electrons of the source gas molecules were excited into their electronic excited states. SiH_4_ excited states are usually dissociating states from which dissociation occurs to SiH_3_ (long life time species), SiH_2_, SiH, Si (short life time species), H_2_ and H [9]. The species control of SiH_4_ and H_2_ dissociation is crucial to the film quality and the growth rate. SiH_3_ is a dominant and mainly expected film precursor for the polycrystalline Si growth. However, the short life time species, which causes the deterioration of structural properties in the resulting films, is highly dissociated at the high electric power and the low SiH_4_ flow rate conditions. In the case of H_2_, the hydrogen radical enhances precursor surface diffusion by the surface hydrogen coverage, acts as an etchant of weak Si–Si bonds, incorporates into the existing crystalline site, all of which result in the promoting the growth of the crystalline phase [10,11,12]. Accordingly, the applied electric power and the electron temperature of the plasma should be controlled to maximize the SiH_3_ portion in SiH_4_ dissociation, although a high electric power and, thus a high plasma density, are needed to enhance crystalline quality by H_2_ dissociation. By inserting a showerhead and thus dividing the space into two plasma discharge spaces with different electron temperatures, source gas decomposition can be controlled. Namely, H_2_ dissociation can be accelerated through injection at the high electron temperature region, and SiH_4_ can be injected at the low electron temperature region to encourage the dissociation of SiH_3_ and prevent the dissociation of the short life time species.

**Figure 3:**
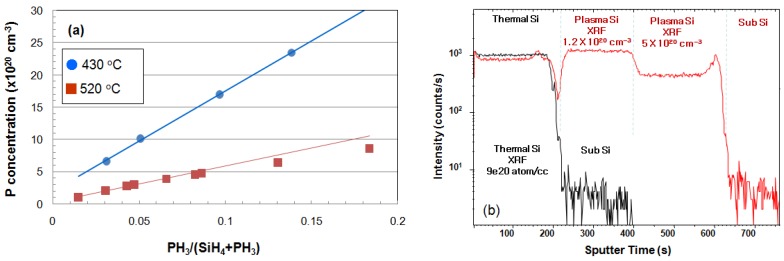
(**a**) Phosphorus (P)-concentration with the PH_3_ flow rate; the blue circles represent the X-ray fluorescence (XRF) results of the Si grown at 430 °C, and the red rectangles represent the XRF results of the Si grown at 520 °C. (**b**) The secondary ion mass spectrometry (SIMS) profile of the Si layer grown by thermal CVD (black) and MW-CVD (520 °C, red).

The doping concentration of the as-grown Si layer measured by XRF and SIMS is represented in Figure 3. In-situ P incorporation during Si growth at 430 °C was linearly increased with the PH_3_ flow ratio without saturation up to 2.5 × 10^21^ cm^−3^, thus indicating that the dopant incorporation was not saturated and further higher doping is possible. However, at 520 °C, the P-concentration was lower than that of 430 °C, and the saturation of dopant incorporation started at the PH_3_ flow ratio of 0.1. PH_3_ initially molecularly adsorbed on Si (1 0 0) at room temperature and gradually converted to PH_2_ at 350 °C [13]. Additionally, PH_3_ decomposed to PH_2_ before the surface adsorption in the MW-CVD system, because PH_3_ was injected into the high electron temperature region (upper side of chamber). On the contrary, SiH_4_ was injected at the low electron temperature region, and the absorption of decomposed SiH_3_ was limited by the thermal reaction. The growth temperature could be used up to 520 °C, but the growth rate was so much higher than the H etch rate that it was hard to see the effect of hydrogen. At 430 °C, a hydrogen crystallinity effect and the selectivity of the epitaxy growth were obtained. Thus, we could obtain a higher P-concentration by MW-CVD compared to thermal CVD, and the higher doping was achieved by using a lower growth temperature. It has the non-pattern wafer used for thermal CVD and MW-CVD experiments under this growth condition. The P-concentration measured by XRF was confirmed by SIMS. SIMS was used for the relative comparison only as a qualitative analysis method. Figure 3b shows the depth profile of the P-concentration in the Si layer grown by the thermal CVD (black) and the Si layer grown by MW-CVD at 520 °C. The red line shows that the P concentration measured by XRF was well matched in the range of 5 × 10^20^–1.2 × 10^21^ cm^−3^, and the stiff change of doping concentration was achieved in MW-CVD. The peak appearing at the interface of sub Si was the MW plasma initial ignition effect, where the MW plasma was ignited higher at 700 mTorr than the process pressure.

**Figure 4:**
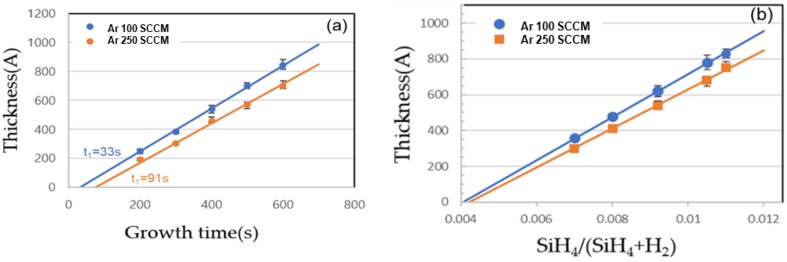
The thickness and the linear plot of the polycrystalline Si grown at 520 °C on 100-nm-thick SiO_2_ with (**a**) growth time (SiH_4_ flow rate of 7 SCCM) and (**b**) SiH_4_ flow ratio (growth time of 300 s).

The deposition kinetics of the Si grown by MW-CVD at 520 °C is represented in Figure 4. The *x*-intercepts, called the incubation times (*t*_I_), at the growth on SiO_2_ 33–91 s (Figure 4a) with the Ar flow rate of 100–250 SCCM indicates a possibility of selectively growth on the SiO_2_ patterned Si wafer. According to Schroeder et al. [14], reactive atomic hydrogen has a major effect on the nucleation and the growth processes, suggesting that the hydrogen atoms adsorbed on Si-like sites on SiO_2_ can effectively block the nucleation of Si. However, in terms of promoting selective area growth, coincident atomic hydrogen reduces the epitaxial growth rate, essentially offsetting any increase in the *t*_I_ for growth on SiO_2_. Though we fixed the H_2_ flow rate at 1000 SCCM, similar results were obtained by varying the Ar flow rate at 100–250 SCCM, because the plasma density was proportional to the Ar flow rate, resulting in an increase of H_2_ dissociation. This reactive atomic hydrogen radical also etched the Si atom. Granata et al. [15] proposed that the H_2_ plasma selectively etches Si toward silicon nitrides hard masks. Additionally, depending on the power density and the temperature, an amorphous-to-crystalline transition can approached. In our system, the hydrogen etch process was activated at an SiH_4_ flow ratio below 0.004, as shown in Figure 4b. Using the H_2_ plasma that simultaneously promotes the deposition and the etch processes, we set up the cyclic selective growth technique by controlling the SiH_4_ flow rate—specifically, at the deposition step, a high SiH_4_ flow ratio (>0.004) was used within t_I_ at the etching step, and low SiH_4_ flow ratio (<0.004) was used to refresh the dielectric surface by removing Si nuclei. We tried to fill the gBC pattern of the 20 nm pitch DRAM device using the cyclic Si selective growth technique. The pattern size of gBC was 20 × 30 nm^2^, and the height was approximately 200 nm, resulting in an aspect ratio of 10.

**Figure 5:**
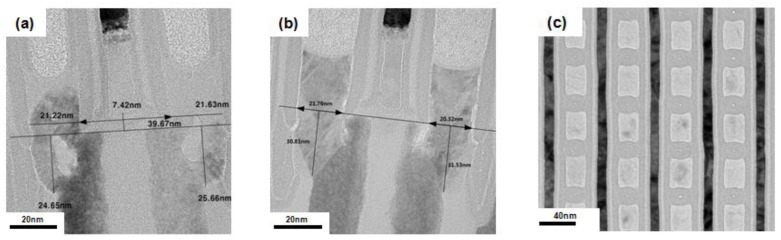
Cross-section transmission electron microscope (X-TEM) images of the gate buried contact (gBC) pattern after Si growth and etch-back. Si is grown by (**a**) thermal CVD with the amino-silane seeding and (**b**) MW-CVD. (**c**) A plan view TEM image of the Si grown by MW-CVD in the gBC pattern.

**Figure 6:**
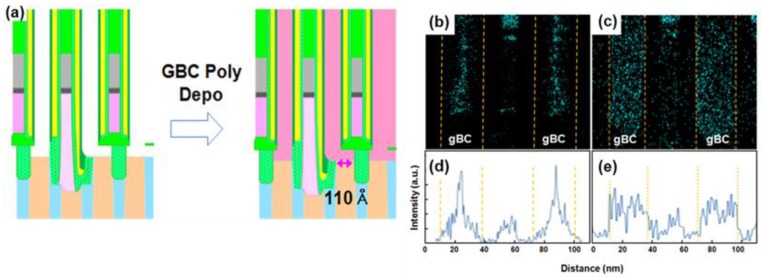
Energy dispersive spectroscopy (EDS) 2D profiles and line profiles of P doping concentration in the Si grown by (**a**) the gBC L-shaped profile, (**b**,**d**) thermal CVD, and (**c**,**e**) MW-CVD in the gBC pattern.

Figure 5a shows a conventional X-TEM image of a 20 nm pitch DRAM gBC pattern after Si gap-filling and etching back (including additional thermal nitride deposition and etching-back). Though the amino-silane was used for uniform nucleation before thermal CVD growth [1], there were large voids near the interface between the active Si and the thermal grown Si, because Si was simultaneously grown from the bottom active Si and the side wall dielectric. The TEM image also shows that the thermal grown amorphous Si at the contact area was crystallized, thus forming the CEG due to the thermal process (~800 °C) of the following nitride deposition. However, the dispersion of the contact area and the CEG height resulted in the widespread contact resistance distribution of etch cells. On the contrary, there were no voids and seam formations at the case of the Si grown by MW-CVD (X-TEM shown in Figure 5b). The CEG is formed in all of the contact area. Additionally, although there was no additional thermal energy after the “etch-back” process, we could observe that the all the remaining Si was crystallized. Figure 5c shows a plan view of the TEM image of the Si grown by MW-CVD. In the large area, the gBC pattern was fully filled by polycrystalline Si, thus indicating the bottom-up filled Si and the dielectric side wall were well-adhesive without slit void formations. The doping distribution of the gap-filled Si in the gBC grown by conventional thermal CVD and MW-CVD is compared in Figure 6 by EDS. A gBC channel which filled the Si grown by conventional thermal CVD had a non-uniform P-concentration due to the undoped amino-silane seeding layer and the doping concentration degradation at the initial stage of Si deposition. The two-dimensional profile shows that P-atoms were concentrated in the center area of the gBC Si. The line profile also shows that the P-concentration of center area was five times more than that of the edge area, resulting in the reduction of the electrical contact area and, thus, the increase of contact resistance. However, the high doping concentration with a uniform doping distribution was achieved in the Si grown by MW-CVD, resulting in a large electrical contact area which was not affected by line pitch distribution.

**Figure 7:**
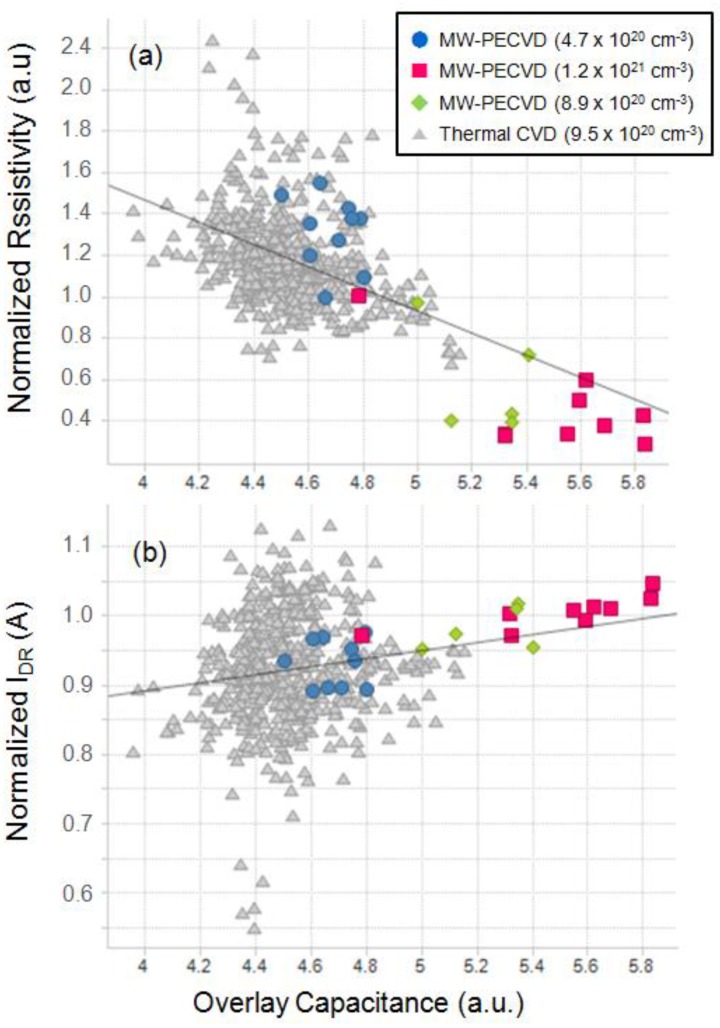
(**a**) The gBC resistivity (*R_C_*) and (**b**) on-current (*I_DR_*) of the 20 nm pitch DRAM (Dynamic Random Access Memory) device with various MW-CVD growth conditions.

Figure 7 shows the electrical characteristics results of 20 nm pitch DRAM device with the Si gap-filled gBC grown by thermal CVD and MW-CVD. The gBC resistivity (*R_C_*) was reduced, and the on-current (*I_DR_*) thus increased with the doping concentration range of 4.7–8.9 × 10^20^ cm^−3^. Additionally, although the Si grown by MW-CVD had a low doping concentration of 4.7 × 10^20^ cm^−3^, similar *R*_C_ and *I_DR_* values were obtained compared to the Si grown by the thermal CVD (9.5 × 10^20^ cm^−3^). The overlay capacitance determined by cell structure and the doping concentration also increased with the doping concentration of the Si grown by MW-CVD, and a similar value of 4.7 × 10^20^ cm^−3^ was reached with conventional device. The bottom-up Si gap-filling by MW-CVD suppressed seam and void formation and made a uniform P-concentration in the contact, resulting in an increase of electrical contact area, and, thus, a low *R_C_* and the high *I_DR_*.

## 4. Conclusions

MW-CVD with an RLSA was firstly developed to grow polycrystalline Si. We successfully grew Si at a low temperature (~430 °C) with a high P doping concentration (>2.5 × 10^21^ cm^−3^) by inserting a showerhead that allowed us to control source gas decomposition. After optimizing the selective growth condition on bare Si and a 100-nm-thick oxide wafer, we tried bottom–up Si growth on the 20 × 30 nm^2^ gBC pattern. A uniform doping distribution and an excellent gap-fill ability without seam and void formation was achieved, thus resulting in a low contact resistivity and a high transistor on-current.
